# Methyl jasmonate root secretion improves low phosphorus tolerance in apple rootstock

**DOI:** 10.1093/plphys/kiaf357

**Published:** 2025-08-18

**Authors:** Catherine Freed

**Affiliations:** Department of Biochemistry, University of Wisconsin-Madison, Madison, WI 53706, USA; Assistant Features Editor, Plant Physiology, American Society of Plant Biologists

Phosphorus (P), while a critical macronutrient that promotes crop growth, survivorship, and yield, is limited and nonrenewable (Cordell and White 2011; Herrera-Estrella and López-Arredondo 2016). While farmers can temporarily provide P by applying fertilizers, this depletes P mines and poses significant environmental risks, such excessive accumulation of P in soil and watersheds that lead to algal blooms. Despite the addition of fertilizers, P is often inaccessible in agricultural land due to minerals in the soil forming insoluble complexes with P (Lambers et al. 2015; Abel 2017). Understanding crop responses to low phosphorus (LP) stress is crucial for enhancing plant resilience and P use efficiency in agriculture.

Plants have developed multiple molecular and physiological mechanisms to survive LP stress by reprioritizing growth patterns to increase P uptake and redistribution from cells. (Cho et al. 2021; Madison et al. 2023). While these mechanisms have been well explored in model species, how LP impacts apple trees is poorly understood. In the apple industry, scions are attached to rootstocks to bolster productivity and stress tolerance. Rootstocks must be efficient at absorbing and transporting water and nutrients to the scion. Therefore, understanding how rootstocks respond to LP will improve apple resilience and productivity.

Recently in *Plant Physiology*, Zhao et al. 2025 explored the mechanisms of LP tolerance in apple (*Malus domestica* Borkh.) rootstocks using a multi-omics approach. First, they examined the progeny of an LP-tolerant variety rootstock and an LP-sensitive variety rootstock using bulked segregant analysis sequencing and RNA sequencing. By combining these analyses, they identified 471 genes associated with LP tolerance, including candidate genes involved in P signaling as well as numerous diverse transcription factors, suggesting that the rootstock response to LP is likely regulated by multiple biological processes.

Zhao et al. also examined metabolites from root exudates of tolerant and sensitive parent rootstocks under LP. Their analysis revealed that under LP conditions, the tolerant variety had a significant enrichment of secreted methyl jasmonate (MeJA), a molecule critical for plant defense, compared to the sensitive variety. Notably, past work links MeJA as a promoter of root hair formation (Zhu et al. 2006; Uddin et al. 2013; Zhu et al. 2015), an important plant trait for increasing P uptake (Cho et al. 2021 ; Madison et al. 2023 ). The authors also observed higher expression of MeJA biosynthetic genes in the tolerant variety and observed a negative correlation in expression with HD-Zip transcription factor, MdHB52. Silencing *MdHB52* increased plant P accumulation and MeJA secretion compared to control plants, while overexpressing *MdHB52* yielded plants with lower MeJA secretion and lower P accumulation. This indicates that MdHB52 likely regulates P absorption in apple rootstocks by inhibiting MeJA secretion.

The authors identified an SNP variation between sensitive and tolerant varieties within the *MdHB52* promoter region that inhibited *MdHB52* expression compared to the promoter without the variation. The authors also found that silencing *MdHB52* increased MeJA synthesis genes. These data suggest that inhibiting *MdHB52* in the tolerant rootstock increased expression of MeJA genes and MeJA root secretion, while increased *MdHB52* expression in sensitive rootstock had the opposite impact on MeJA synthesis (Fig.).

**Figure.**
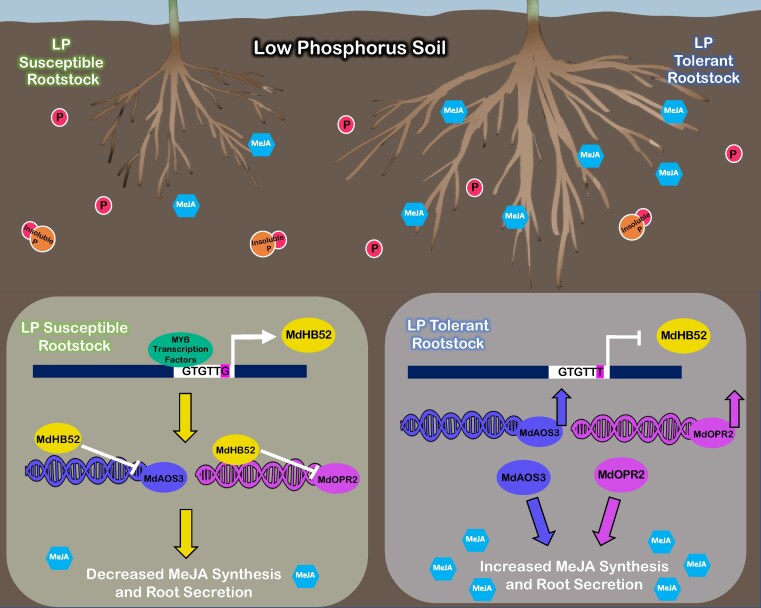
A model of LP-sensitive and LP-tolerant apple rootstocks grown under LP conditions, adapted from Zhao et al. 2025. In LP-sensitive rootstocks (left), expression of *MdHB52* is mediated by MYB transcription factors under LP conditions. MdHB52 represses MeJA biosynthetic genes *MdAOS3* and *MdOPR2* and leads to a lower MeJA synthesis and secretion from roots. In LP-tolerant rootstocks (right), *MdHB52* expression is repressed due to an SNP variation in the promoter region and, as a consequence, promotes expression of *MdAOS3* and *MdOPR2* and increases MeJA synthesis and its secretion from roots. This in turn leads to increased P absorption under LP conditions. Hexagons represent MeJA, spheres represent plant-available P, and two combined spheres represent plant-unavailable or insoluble P in the soil.

This study offers novel mechanistic insights on how a PT rootstock variety confers greater resistance to growing under LP stress conditions. The tolerant rootstock inhibits *MdHB52* expression, increasing MeJA biosynthetic gene expression, MeJA root secretions, and P accumulation. By combining multiple sequencing datasets, the authors were able to identify unique targets and explore their impact on root exudates and P absorption under LP conditions. Understanding how MeJA promotes root hair formation and P absorption in concert with other factors merits further investigation. Expanding our understanding of enhancing root growth under LP conditions is critical for enhancing P use efficiency and resilience in crops.

## Data Availability

No new data were generated or analyzed in support of this research.
